# A Double Threat in an Immunocompromised Patient: Coinfection With Pneumocystis jirovecii Pneumonia and Cytomegalovirus in Stomach Cancer

**DOI:** 10.7759/cureus.96628

**Published:** 2025-11-11

**Authors:** Rashmi R Pattanayak, K Smita Reddy, Biswabikash Mohanty

**Affiliations:** 1 Critical Care Medicine, Utkal Hospital Private Limited, Bhubaneswar, IND; 2 Anesthesiology, All India Institute of Medical Sciences, Bhubaneswar, IND

**Keywords:** acute respiratory distress syndrome (ards), cytomegalovirus (cmv), infections in immunocompromised, interstitial pneumonia, pneumocystis pneumonia (pcp)

## Abstract

*Pneumocystis jirovecii* pneumonia (PCP) and cytomegalovirus (CMV) present significant threats as opportunistic infections among immunocompromised patients. The combination of these two infections can lead to dire clinical scenarios, particularly in those with weakened immune systems. We document a case of a patient battling advanced stomach cancer who was admitted with alarming symptoms, including persistent fever and severe respiratory distress. Having undergone multiple chemotherapy and immunotherapy cycles, patient's condition had deteriorated significantly. Initial management involved high-flow nasal cannula support; however, patient's respiratory status soon declined, progressing to acute respiratory distress syndrome, which necessitated the use of mechanical ventilation. A thorough bronchoscopy was conducted, and the subsequent bronchoalveolar lavage revealed a dual infection, confirming the presence of both PCP and CMV. Patient received a combination of trimethoprim-sulfamethoxazole and ganciclovir, specifically targeting the identified infections. To combat the hypoxia exacerbated by PCP, patient received a steroid. Remarkably, the patient was eventually weaned off the ventilator, marking a significant turn in their clinical course.

## Introduction

*Pneumocystis jirovecii* pneumonia (PCP) is a common opportunistic respiratory infection that affects patients with human immunodeficiency virus (HIV) and other immunocompromised conditions, such as malignancies [1]. Cytomegalovirus (CMV) pneumonia is another opportunistic infection that can arise in these patients during periods of severe immunosuppression [2]. *Pneumocystis jirovecii* spreads between humans via the airborne route with strong tropism for the lungs, leading to severe PCP. Symptoms like dry cough, dyspnea, low-grade fever, and tachypnea characterize PCP. PCP spreads through the air, from person to person. Some healthy adults can carry the Pneumocystis fungus in their lungs without having symptoms, and it can spread to other people, including those with weakened immune systems [3]. The pulmonary manifestations of CMV infection can range from a dry cough to severe interstitial pneumonia, with patients presenting with diffuse pulmonary infiltrates that resemble a ground-glass appearance. The diagnosis of CMV pneumonia is based on radiological patterns and serology (CMV IgM antibody) or polymerase chain reaction (PCR) [4]. This case report describes an immunocompromised patient who experienced a coinfection with both PCP and CMV, which is a unique presentation. The patient received the recommended therapies for each infection and achieved a favorable outcome.

## Case presentation

A 62-year-old man with advanced metastatic stomach cancer was admitted to the hospital with a fever lasting three days, respiratory distress lasting four days, and generalized weakness lasting eight to 10 days. He had received chemotherapy, paclitaxel, and ramucirumab 15 days prior to admission. Before that, he had undergone eight cycles of capecitabine and oxaliplatin-based chemotherapy.

**Figure 1:**
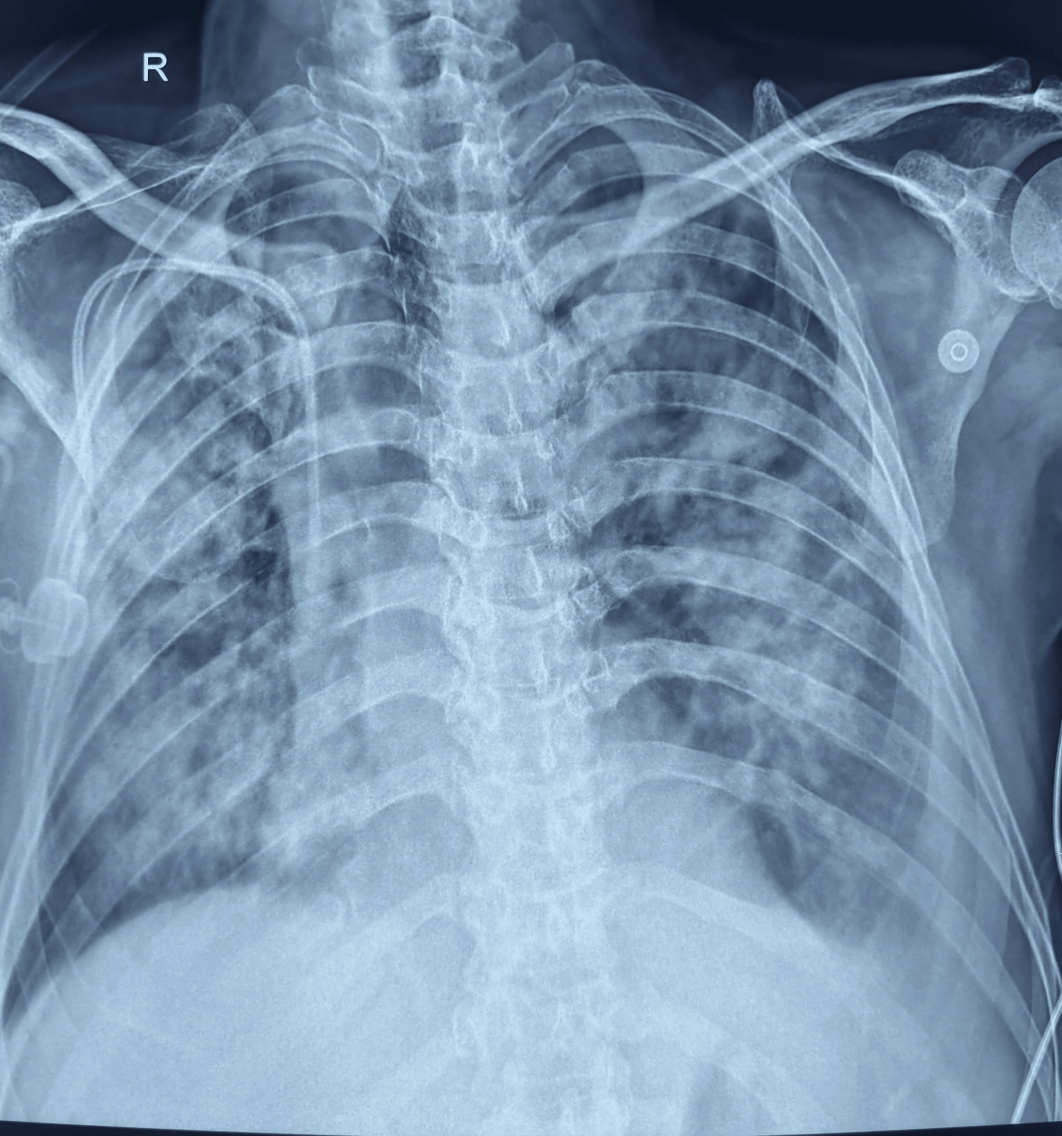
Shows bilateral opacity suggestive of acute respiratory distress syndrome.

Because of respiratory distress, the patient was transferred to the intensive care unit (ICU). A point-of-care ultrasound revealed bilateral B-lines, consolidation in both lung fields, and bilateral pleural effusion (left greater than right), with good left ventricular contractility and no regional wall motion abnormalities. Arterial blood gas analysis indicated hypoxemic respiratory failure (PO_2_ of 60 mm Hg) while on six liters of oxygen. Given his tachypnea and oxygen requirement, he was placed on a high-flow nasal cannula (FiO_2_ of 60% at a flow rate of 60 liters). Blood cultures were sent, and broad-spectrum antibiotics were initiated. A chest X-ray was suggestive of acute respiratory distress syndrome (ARDS) (Figure 1).

Table 1 shows the result of initial investigations. There was a trend towards lymphopenia. Both β-d-glucan (BDG) and lactate dehydrogenase (LDH) were elevated subsequently.

**Table 1:** Lab results of initial investigations. TLC: total leukocyte count; SGOT: serum glutamic oxaloacetic transaminase; SGPT: serum glutamic pyruvic transaminase; LDH: lactate dehydrogenase.

Lab parameters	Reference	Day 1	Day 3	Day 5	Day 7	Day 9
Hb (gm/dl)	14-18	10.4	9.9	9.7	10.7	10.5
TLC (10^9^/L)	4-11	7.91	7.54	13.09	13.55	14.41
Neutrophil (%)	30-70	79.8	90	86.8	90.6	88.8
Lymphocyte (%)	20-40	18.0	6.9	9.7	7.7	8.9
Platelet (10^9^/L)	1.5-4	189	165	254	422	343
Urea (mg/dl)	7-20	23.9	24.8	47.9	45	46
Creatinine (mg/dl)	0.8-1.3	0.84	0.81	0.7	0.61	0.7
SGOT (U/L)	0-35	22.5	_	_	_	34
SGPT (U/L)	4-36	21	_	_	_	30.6
Bilirubin (mg/dl)	0.3-1	0.5	_	_	_	0.7
Albumin (gm/dl)	3.5-5.5	2.86	_	_	_	2.89
Procalcitonin (ng/ml)	Less than 0.1	0.30	_	_	0.63	_
LDH (u/l)	140 to 180	_	_	836	_	_
β-d-glucan (pg/ml)	Less than 60	_	_	120.1	_	_
D-dimer (ng/ml)	Less than 500	_	1694.2	_	_	_

As the respiratory distress worsened, patient was intubated and required an FiO_2_ of 90% to maintain a PO_2_ of 70 mm Hg, indicating severe ARDS. A bronchoscopy was performed, and a bronchoalveolar lavage (BAL) sample was sent for Gram stain, fungal stain, culture, and polymerase chain reaction (PCR) testing. Empirically, oseltamivir 75 mg twice daily was started for suspected influenza pneumonia. Patient was then placed in prone ventilation for two cycles, during which his oxygenation improved to a PO_2_ of 70 mm Hg on FiO_2_ of 50%.

**Figure 2:**
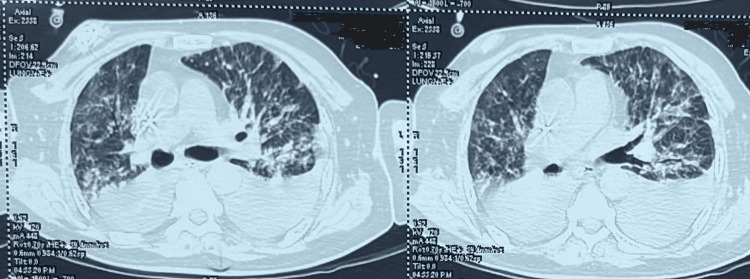
Showing bilateral ground-glass opacity with bilateral pleural effusion and bilateral consolidation suggestive of ARDS. ARDS: acute respiratory distress syndrome.

The BAL sample PCR results indicated infections with PCP, CMV, and *Klebsiella pneumoniae*. Treatment was initiated with intravenous trimethoprim-sulfamethoxazole (TMP-SMX) at a dose of trimethoprim 15 mg/kg and sulfamethoxazole 75 mg/kg in three divided doses for PCP, and ganciclovir at 5 mg/kg twice daily for CMV pneumonia. Oseltamivir was stopped. Furthermore, antibiotics were optimized based on culture sensitivity for *Klebsiella pneumoniae*. The BAL fluid was negative for fungi, but the culture did grow *Klebsiella pneumoniae* (multidrug-resistant). Gomori-Grocott methenamine silver nitrate staining for PCP was done and returned a negative result. To address the hypoxia associated with PCP, oral prednisolone at 40 mg twice daily was administered for five days and then 40 mg once daily for five days, as patient was having persistent hypoxia with alveolar-arterial gradient of 45 mm Hg. After proning and steroid therapy, hypoxia improved, and the alveolar-arterial gradient also decreased to 15 mm Hg. A high-resolution computed tomography (HRCT) scan of the chest suggested bilateral consolidation (Figure 2).

**Figure 3:**
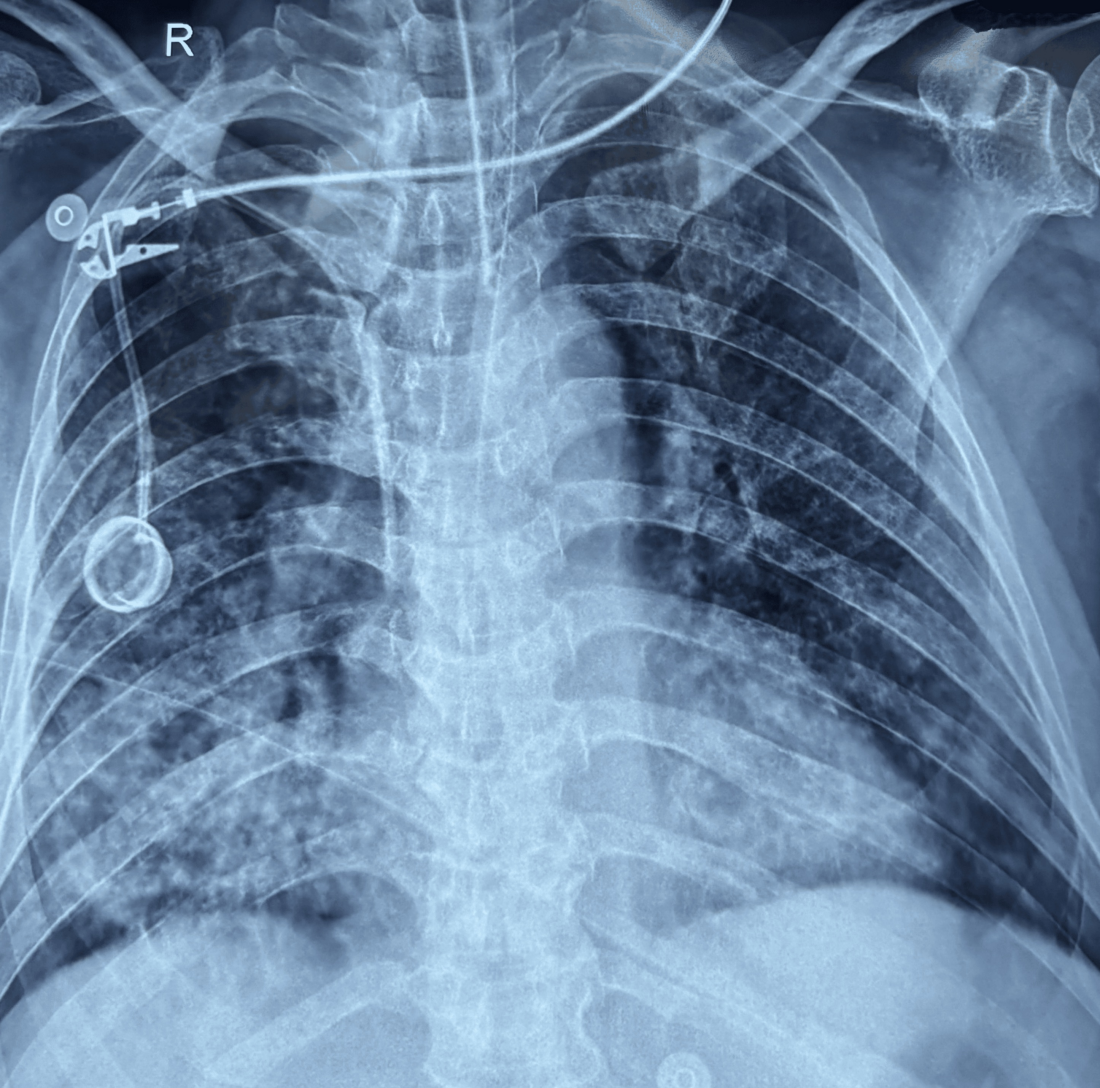
Chest x-ray suggestive of ARDS resolution. ARDS: acute respiratory distress syndrome.

Over time, the patient's oxygenation improved. In Figure 3, the chest x-ray was suggestive of ARDS resolution.

After nine days of mechanical ventilation, patient was extubated. A serum CMV titer was sent and returned undetectable. Patient was treated with ganciclovir and TMP-SMX for a total of 14 and 21 days, respectively, for CMV pneumonia and PCP. The total hospital stay was 28 days, after which patient was discharged on oral antibiotics. Upon follow-up two months later, patient reported doing well and subsequently received chemotherapy as the oncologist advised.

## Discussion

This case report presents a rare instance of PCP and cytomegalovirus pneumonia in a patient with advanced stomach cancer. To our knowledge, this is the first report documenting the concurrent occurrence of PCP and CMV infection in stomach cancer patients, making it unique. Although advances in cancer chemotherapy have extended treatment durations, these extended treatments significantly increase infection risk due to cumulative immunosuppression. A key takeaway from this case report is the necessity of considering prophylactic administration of TMP-SMX for patients at high risk of developing PCP or ganciclovir for patients at high risk of developing CMV infections. Additionally, this report highlights the importance of early diagnosis and pre-emptive treatment when an infection is suspected.

Incidents of PCP in cancer patients are relatively rare. One study found that the cumulative incidence of PCP in patients with Hodgkin lymphoma receiving chemotherapy was 6.2% after one year [1]. Another study reported an overall incidence of 0.6% in breast cancer patients undergoing anthracycline-based chemotherapy [5].

CMV pneumonia is a serious complication with varying incidence rates among cancer patients. Higher risks are observed in patients with hematologic malignancies, particularly following stem cell transplantation, and in patients receiving immunosuppressive therapies, such as checkpoint inhibitors. Incidence of CMV pneumonia in cancer patients can differ based on cancer type, treatment received, and patient's immune status. It can range from a few percent to more than 10% in certain populations. CMV pneumonia can lead to significant morbidity and mortality, especially in immunocompromised individuals, if not diagnosed and treated early [2].

Coinfection with PCP and CMV is extremely rare in cancer patients. However, it is more common in HIV-infected individuals who have severe immunosuppression. A CD4 count below 200 cells/mm³ significantly increases the risk of PCP, whereas CMV infection typically occurs when the CD4 count drops below 50 cells/mm³.

Definitive diagnosis of PCP requires detection of PCP in a respiratory specimen, either by microscopic examination or by PCR. In our case, microscopic examination was negative, but the PCR from BAL fluid was positive for PCP. In this case, BDG (120.1 pg/ml) and LDH (836 U/l) levels exceeded the reference ranges significantly, consistent with reports indicating elevated serological markers in patients positive for PCP [6]. The highly positive predictive value of BDG, with a cutoff of 33.5 pg/ml, and the CT findings supported the diagnosis of PCP in this case.

CMV pneumonia can be diagnosed by detecting the virus in serum or respiratory samples, such as BAL or tracheal aspiration [6]. Little evidence is available regarding the incidence of CMV disease in patients with solid cancers. The latest data show that approximately 50% of patients with CMV PCR positivity developed clinically relevant CMV viremia and would require specific therapy. In the clinical arena, CMV reactivation is an important differential diagnosis in these patients' workup, but guidelines for management on this subject are not yet available. CMV reactivation should be considered during differential diagnosis for patients with a severe decline in lymphocyte counts who are receiving chemoradiotherapy or immunochemotherapy with lymphocyte-depleting or blocking agents. Monitoring of CMV reactivation followed by the implementation of pre-emptive strategies or the establishment of early antiviral treatment improves the prognosis and reduces these patients' morbidity and mortality [7]. Quantitative real-time PCR (qRT-PCR) can be used to measure viral loads in blood and BAL fluid [8]. Lung biopsy histopathology is considered the gold standard for diagnosing pulmonary CMV infections, with the presence of CMV inclusion bodies (owl's eye) in biopsy specimens confirming lung infection. However, the diagnostic yield of lung biopsy in lung CMV infections can vary; inclusions may not always be visualized. In the case study, BAL fluid PCR yielded high CMV and PCP titers.

The relationship among chemotherapy, corticosteroids, and the risk of PCP in patients with solid tumors warrants further investigation. Reports on other cancers have indicated that high-dose chemotherapy increases the risk of PCP [9]. In the current case, the 25 cycles of chemotherapy every two weeks and prolonged intermittent use of steroids may have caused lymphopenia, contributing to the subsequent occurrence of PCP.

PCP pneumonia is primarily treated using antibiotics, with TMP-SMX being the most common choice. The recommended dosage is 15-20 mg/kg of trimethoprim and 75-100 mg/kg of sulfamethoxazole in a divided dose administered three to four times a day intravenously. This can be converted to oral tablets once the patient improves for a total of 14-21 days. For patients with moderate to severe PCP, especially those showing low oxygen levels (PaO_2_ <70 mmHg and an A-a gradient >35 mmHg), adjunctive corticosteroids may be beneficial. The suggested regimen for corticosteroids is prednisone at 40 mg twice daily for five days, followed by 40 mg once daily for an additional five days [10].

CMV pneumonia in cancer patients is generally treated with antiviral medications, most commonly ganciclovir or valganciclovir. The recommended dose of ganciclovir is 5 mg/kg, administered twice daily. The treatment duration can vary, particularly for patients undergoing chemotherapy, and may involve either intravenous or oral administration of the medication. In some cases, foscarnet may be used as an alternative or alongside ganciclovir, especially for patients experiencing myelosuppression. For patients who have undergone hematopoietic stem cell transplantation, the treatment typically includes induction therapy for 21 to 28 days, followed by maintenance therapy for four weeks. In chemotherapy patients, the treatment duration may range from three to 30 weeks. Monitoring the CMV viral load through PCR testing is crucial, particularly in patients with solid tumors, because it helps guide treatment and evaluate the response. In our case report, the BAL fluid PCR test was positive for Cytomegalovirus. As a result, ganciclovir was initiated at a dose of 5 mg/kg intravenously, twice daily. Following this treatment, the serum CMV titer became undetectable. Consequently, ganciclovir was administered intravenously for seven days, followed by oral valganciclovir at a dose of 450 mg twice daily for an additional seven days [8].

In immunocompromised patients, coinfection with PCP and CMV is associated with a poorer prognosis compared to PCP alone, especially in HIV-negative individuals. This coinfection can lead to increased mortality, longer stays in the ICU, and a higher incidence of complications such as ARDS and the necessity for mechanical ventilation. Ekren et al. [11] found that ARDS and the need for mechanical ventilation were significantly more common in the CMV coinfection group (P = 0.019 and P = 0.031, respectively), and the duration of ICU stays was also longer (P = 0.006). In univariate analyses, the mortality rate at 30 days was higher in the CMV coinfection group compared to that group with PCP alone, at 78.6% and 46.7%, respectively (P = 0.046). In multivariate analyses, mortality was found to be independently associated only with the presence of ARDS (odds ratio: 6.22, 95% confidence interval). Meanwhile, its association with CMV coinfection was no longer considered significant (odds ratio: 2.6, 95% confidence interval, P = 0.257).

In summary, CMV coinfection significantly worsens the prognosis for patients with PCP, particularly in HIV-negative individuals. Early diagnosis and treatment of both infections are essential for improving outcomes [12].

## Conclusions

PCP‑CMV coinfection should be considered in patients with severe hypoxemia and an immunocompromised state, particularly cancer patients undergoing chemotherapy and immunotherapy. Appropriate and early treatment reduces the mortality and morbidity associated with the coinfection. Prophylactic treatment with trimethoprim-sulfamethoxazole and ganciclovir may be initiated early in cancer patients who present with hypoxia and lymphopenia.
